# The impact of tumor associated macrophages on tumor biology under the lens of mathematical modelling: A review

**DOI:** 10.3389/fimmu.2022.1050067

**Published:** 2022-11-10

**Authors:** Pejman Shojaee, Federica Mornata, Andreas Deutsch, Massimo Locati, Haralampos Hatzikirou

**Affiliations:** ^1^ Centre for Information Services and High Performance Computing, Technische Universität (TU) Dresden, Dresden, Germany; ^2^ Leukocyte Biology Lab, IRCCS Humanitas Research Hospital, Rozzano, Italy; ^3^ Department of Medical Biotechnologies and Translational Medicine, Universitàdegli Studi di Milano, Milan, Italy; ^4^ Mathematics Department, Khalifa University, Abu Dhabi, United Arab Emirates; ^5^ Healthcare Engineering Innovation Centre (HEIC), Khalifa University, Abu Dhabi, United Arab Emirates

**Keywords:** tumor-associated macrophage (TAM), tumor-macrophage interactions, tumor microenvironment (TME), mathematical modelling, cancer immunotherapy

## Abstract

In this article, we review the role of mathematical modelling to elucidate the impact of tumor-associated macrophages (TAMs) in tumor progression and therapy design. We first outline the biology of TAMs, and its current application in tumor therapies, and their experimental methods that provide insights into tumor cell-macrophage interactions. We then focus on the mechanistic mathematical models describing the role of macrophages as drug carriers, the impact of macrophage polarized activation on tumor growth, and the role of tumor microenvironment (TME) parameters on the tumor-macrophage interactions. This review aims to identify the synergies between biological and mathematical approaches that allow us to translate knowledge on fundamental TAMs biology in addressing current clinical challenges.

## 1 Introduction

Macrophages are a heterogeneous population of immune cells and are present in every tissue, where they play a crucial role in maintaining tissue integrity and homeostasis, displaying great functional diversity. Macrophages are critical for physiological functions, such as wound healing, innate immune responses, and are involving in a plethora of pathological contexts, including cancer. According to their inflammatory activity, macrophages have been classified into M1 and M2 phenotypes. Though this classification has limitations, it has been instrumental to define the concept of distinct macrophage functional phenotypes and still provides a useful conceptual framework to dissect the role of macrophages in different biological settings. TAMs exhibit complex behavior and dual functions in their interactions with neoplastic cells (1, 2). During the initial stages of tumor development, macrophages can either directly promote anti-tumor responses by killing tumor cells or indirectly by recruiting and activating other immune cells. As genetic changes accumulate within tumors, TAMs begin to exhibit immunosuppressive pro-tumor properties that promote tumor progression, angiogenesis, metastasis, and resistance to therapy. For all these reasons, targeting TAMs has emerged as a prominent strategy for cancer therapy. In this regard, many efforts have been invested in developing various experimental models to improve understanding of TAMs and tumor cell interactions and their potential translation to the clinics.

Mathematical models were developed aspiring to perform in silico experiments, to formulate and evaluate biological assumptions, and predict tumor dynamics (3–6). These models have been useful in assessing treatment strategies of different modalities, such as chemotherapy and radiotherapy, and against the tumor progression (7–9). Recently, a plethora of mathematical models were developed to investigate the role of immune cells in the tumor microenvironment (TME) (10–14). We reviewed mathematical models that investigated the role of macrophages in the context of tumor growth and treatment. In this regard, we presented the articles that consider the macrophages as the drug carriers or as the TAMs in the TME. Early models mainly considered the inhibitory effect of macrophages for a model of tumor growth. However, in the most recent papers, the protumoral effect of macrophages was considered and analyzed (15, 16). In section two, we reviewed the biology of TAMs regarding the origin, plasticity, role in tumor progression, and therapeutic effects. In section three, we reviewed the recent mathematical models that considered macrophages as drug carriers, repolarization, plasticity, intracellular signalling, and the interactions between TME elements.

## 2 Biology of TAMS

TAMs are among the most abundant non-neoplastic cells in the TME. They were initially considered to be associated with anti-tumor activities due to their ability to kill tumor cells when activated by cytokines. However, investigators soon highlighted how TAMs promote tumor growth and metastasis in malignant cancers. High infiltration of TAMs has been demonstrated to correlate with poor prognosis and reduced overall survival in different form of cancers, including breast cancer, ovarian cancer, bladder cancer, thyroid cancer, non-small cell lung carcinoma (NSCLC) and Hodgkin’s lymphoma (17). Given the key role played by TAMs in the context of cancer, many efforts have been made to characterize their ontogeny, phenotype and functions in order to develop new therapeutic strategies targeting TAMs (18).

### 2.1 TAMs origin

**Figure 1:**
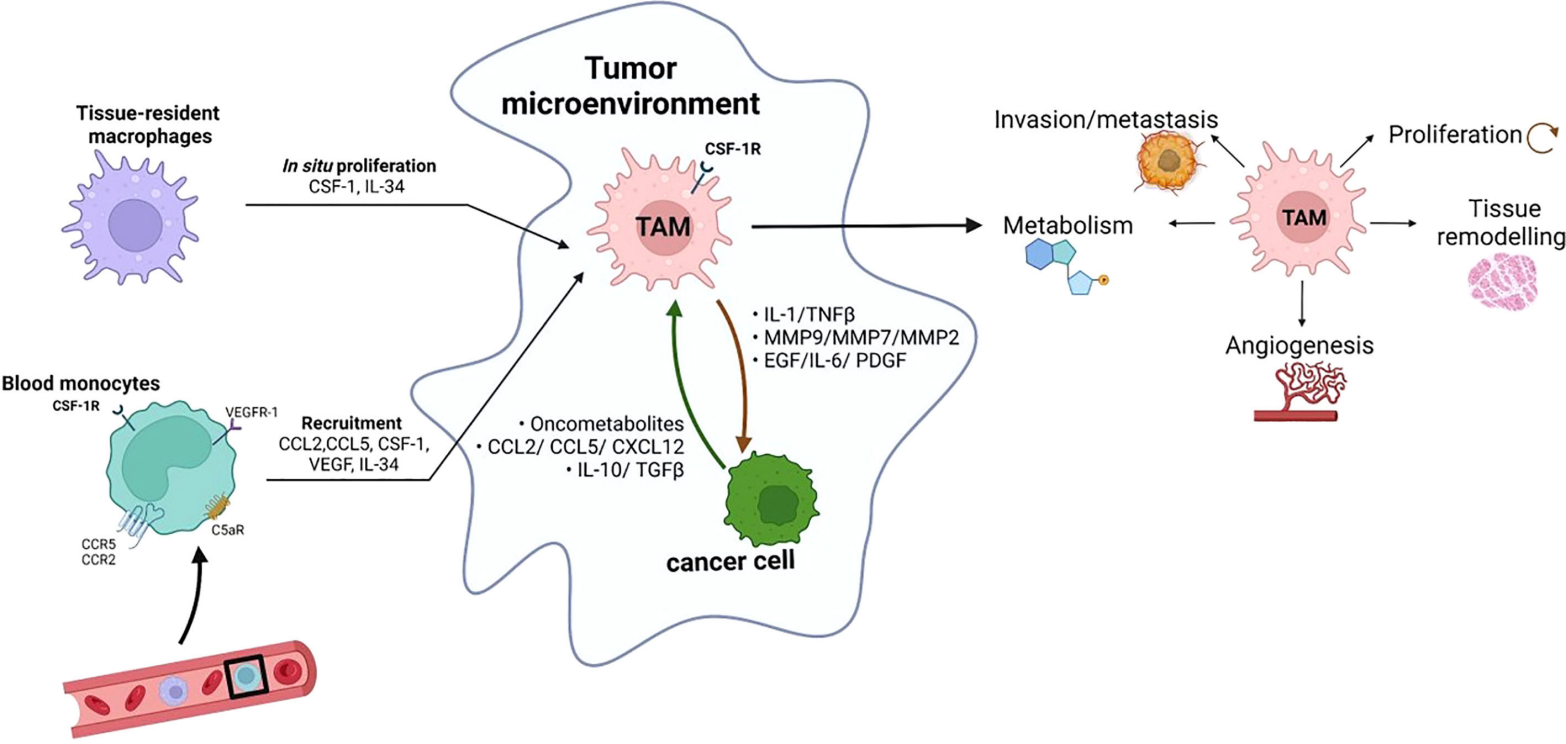
Monocytes in circulation can be recruited by tumors in response to different chemoattractants, including cytokines (such as CSF-1, VEGF and IL-34), chemokines (such as CCL2 and CCL5) and complement components (C5a). The tumor-infiltrating monocytes differentiate into TAMs. In some tumors, *in situ* proliferation can occur and tissue-resident macrophages can contribute to the TAM populations. TAMs influence almost all aspects of tumor-cell biology, including cell proliferation, tissue remodeling, angiogenesis, metabolism, local invasion and metastasis (created with BioRender.com).

TAMs are a complex mixture of heterogeneous cells, whose biology is influenced by many factors including TME composition and disease stage (19). The frequency, location, and diversity of tissue-resident macrophages as well as their functional role is still an open issue. TAMs have long been hypothesized to originate primarily from monocytes recruited to tumor sites by signals released from malignant and non-malignant cells present in the TME. Nowadays, it is known that the profile of tissue-resident macrophages is also affected by nearby neoplastic transformation and they can contribute to the TAM populations (20). TAM proliferation has been observed in some human tumours, but in general, cell division does not provide significant support to the number of TAMs in growing tumors, suggesting that recruitment of circulating progenitors is key in expanding the TAM populations during tumor progression (21). Consistent with this, evidence indicates that a large fraction of TAMs is derived from circulating monocytes (22). The TME dictates the number and type of monocyte and other myeloid cells recruited from the circulation *via* the expression of a wide range of chemoattractants. Bone marrow-derived monocytes are recruited since the early phases of carcinogenesis by chemokines, including CCL2, CCL5, and CXCL12 (23). Once they arrive in the neoplastic environment, monocytes differentiate into mature macrophages, a transition facilitated by tumor-derived hematopoietic growth factors, including monocyte and granulocyte-monocyte colony-stimulating factors (M-CSF and GM-CSF, respectively) (**Figure 1**).

### 2.2 TAMs plasticity

Macrophages are highly plastic. Macrophage polarization is a dynamic process that allows macrophages to adopt a specific phenotype, characterized by specific factors and peculiar biological activities in response to stimuli from the microenvironment and signals arising from different tissues. According to the current classification, macrophages are grouped into two main groups. However, these classes represent the two extremes in a much more complex series of phenotypes that macrophages can assume: the “classical” activation (M1) and the “alternative” activation (M2) (24, 25). This M1/M2 classification is now too limited for TAMs, due to their great diversity; after their recruitment TAMs can acquire the M1 phenotype, expressing MHC-II, CD68, CD80, CD86, and secreting IL6, IL12, IL23, TNFα to exert tumoricidal activities. Alternatively, activated M2 macrophages express CD163, macrophage galactose-type lectin 1(MGL1), MGL2, and MHC-II. They secret IL-10 and TGF- to express a pro-tumor activity. Although, overall, in most TAMs closely resemble M2 macrophages and are reported to be M2-like cells (1, 26).

### 2.3 Role of TAMs in tumor progression

Macrophages may exert both pro-and antitumor activities, according to their functional state of activation within the TME. TAMs may directly promote tumor cell proliferation through the production of growth factors, like epidermal growth factor (EGF) which induces proliferation and supports epithelial-mesenchymal transition in tumor cells (27). Moreover, TAMs support cancer stem cell expansion by producing several mediators, including IL-6 and platelet-derived growth factor (PDGF). Cytokines derived from macrophages such as IL-1 can promote the recruitment and seeding of metastatic cancer cells (19). In addition, TAMs are key players to the neo-angiogenic switch in tumors, a characteristic event that correlates with the transition from benign to malignant tumors since it allows the tumor to expand and metastasize (17). Accordingly, depletion of TAMs by CSF1 expression abrogation has been associated with reduced angiogenesis in preclinical models and restoration of the macrophage population in the tumors rescued the blood vessel phenotype (28). Hypoxia, a major determinant of angiogenesis in cancer, attracts TAMs by the release of hypoxia-induced chemoattractants like CCL-2 and vascular endothelial growth factor (VEGF) (29): TAMs respond to hypoxia by upregulating the expression of inducible transcription factors and their downstream target genes, such as TGF-*β*. As mentioned before, TAMs play a role also in the epithelial-mesenchymal transition by producing soluble factors such as interleukin (IL)-1, IL-18, and tumor necrosis factor-alpha (TNF-α) as well as cathepsins and metalloproteinases (MMP7, MMP9, MMP2) that contribute to the degradation of the extracellular matrix (ECM) (17). TAMs are crucial drivers of immunosuppression in the TME: mediators released by tumor cells and infiltrating lymphocytes, like Th2 cells and Treg cells, and activate an immunosuppressive profile in TAMs (30, 31): as a consequence, immunosuppressive TAMs show a secretory profile characterized by low levels of cytokines promoting adaptive immune responses, as IL-12, IL-18, and high levels of anti-inflammatory/proresolving cytokines such as IL-10 and TGF-*β* (32). Altogether, TAMs acquire an immunosuppressive phenotype that resembles the phenotype of macrophages involved in tissue repair (33). In the context of tumour progression, it is worth to mention the role played by cancer associated fibroblasts (CAFs), that are also an important key factor in TME and have close interactions with TAMs. In particular, CAFs, in contrast to normal fibroblasts, are able to effectively recruit monocytes, through the secretion of chemotactic protein-1 (MCP-1) as well as stromal cell-derived factor-1 (SDF-1) cytokines. CAFs differentiate the recruited monocytes into M2-like macrophages which are capable of exerting their immunosuppressive roles *via* the PD-1 axis. In particular, it has been demonstrated that, in the context of breast cancers, the proliferation rate of tumour cells is increased based on the increased number of CAF-educated monocytes, highlighting the role of the interplay between TAMs and CAFS in promoting tumor cells proliferation and invasion (34, 35).

### 2.4 TAMs as a therapeutic target

The critical role that TAMs play in cancer progression has attracted the efforts of researchers in targeting them by employing various approaches. In general, macrophage-based immunotherapeutic schemes follow three different strategies: reducing the number of TAMs by depleting them or inhibiting the recruitment of their precursors or aiming at re-educating these cells, i.e. reprogramming TAMs from a pro-tumoural to an anti-tumoural state. Early attempts to reduce macrophages’ number relied on bisphosphonates, which have been typically used in the treatment of osteoporosis, with positive effects, especially on bone metastases. Most recently, strategies have aimed at reducing the number of macrophages targeting the CSF1-CSF1R axis: different types of CSF1 receptor inhibitors have been developed and tested in experimental animal models, showing antitumor activity (36–38). Some inhibitors of CSF1R are now being evaluated in clinical studies, in combination with conventional chemotherapy or radiotherapy (39, 40). Another approach to eliminate macrophages is to use chemotherapeutics that selectively targets the monocytic cell lineage, such as trabectedin; in addition to its antineoplastic activity, trabectedin exhibits selective cytotoxicity towards monocytes and macrophages, thus causing a reduction of circulating monocytes and TAMs in tumours (41). However, the main disadvantage of this strategy lies in the fact that systemic and indiscriminate elimination of macrophages may be harmful considering their key role in host defenses and maintenance of homeostasis. Another early approach in reducing the TAMs content adjacent to tumour cells goes through the inhibition of chemoattractants that regulate monocyte recruitment. Chemokines have long been implicated in macrophage accumulation within tumours and their inhibitors, such as anti-CCL2 or CCR2 blockade, have been successfully tested in experimental tumours (42). In particular, specific inhibition of CCL2 with antibodies has been shown to reduce tumour growth and dissemination in different experimental models of prostate, breast, lung, liver cancer, or melanoma (43–46). However, the fact that individual chemokines act on multiple cell types makes the exploitation of these targets as therapeutical strategies challenging. The idea that reprogramming rather than eliminating macrophages might be a better therapeutic approach has fueled the development of many reprogramming strategies, aiming the switching of TAMs from a pro to an antitumoral phenotype. The remarkable functional plasticity of macrophages is the rationale for developing approaches that switch cells from M2-like immunosuppressive TAMs into M1-like immunostimulatory and antitumor cytotoxic effectors. Functional reprogramming of TAMs, or global activation of innate immunity against cancer cells, was tested long ago: for instance, microbial preparations and microorganism-derived molecules such as bacterial muramyl dipeptide can stimulate macrophage-mediated cytotoxicity and have undergone some clinical testing. Intravesical Bacillus Calmette-Guèrin is the only remainder from the “bacterial era of immunotherapy” and it is still used in the treatment of recurrent bladder carcinoma. Alternatively to microbial products, pro-inflammatory cytokines can be used to induce macrophage M1 polarization, supporting tumoural cell killing. For example, IFNγ therapeutic utility has been investigated in patients with ovarian cancer: intraperitoneal IFNγ administration resulted in activation of tumour cytotoxicity and clinical responses (21, 47).

### 2.5 Experimental models to study macrophage-tumour cell interactions

Given the relevant role of TAMs in the context of TME, many efforts have been devoted to develop models that study the interactions between the two cell types. Over the years, many experimental studies of tumour-macrophage interactions evaluated the communication between the two cell populations using co-culture approaches. The majority of such experiments rely on human monocytic cell lines or human peripheral blood mononuclear cells (PBMCs) as a source of macrophages, which can be further polarized *in vitro* using lipopolysaccharide (LPS) and IFNγ or IL-4 to obtain M1 or M2 macrophages, respectively. It is possible to classify co-culture settings into two main categories, namely indirect co-cultures (by “conditioned media” (CM) or transwell devices) or direct cultures. A traditional and widely used approach to study macrophage/tumour cell interactions involves culturing one cell type in the presence of CM from another cell type, typically on a two-dimensional (2D) substrate, such as plastic or glass. This approach has been useful to demonstrate that paracrine signals derived from macrophages, under different phenotypes of activation, can influence key features of cancer cells accordingly to their phenotype of activation: for example, using a colorectal cancer cell as a model, it was shown that M1-conditioned media can reduce tumour cell proliferation, whereas M0 or M2 show no difference (48). Although CM experiments have facilitated our understanding of some of the soluble and molecular mechanisms governing cellular interactions, this culture system is limited in its ability to recapitulate dynamic and reciprocal cell-cell interactions found *in vivo*. Transwell assays have been useful to fill this gap: in this model of co-culture macrophages and cancer cells are separated by a membrane that allows the diffusion of soluble factors between the upper and the lower chamber, while maintaining physical separation between the two cell types. Transcriptomic analysis showed a significant number of up and down-regulated genes in macrophages co-cultured with different cancer cell lines, such as MDA and MB231. Interestingly, cancer cells also showed striking differences, for example up-regulating the expression of tumour progression markers (49). Direct-co-culture studies represent a useful tool to understand the role of recruited macrophages and their influence on the tumour growth and cell death. In one such study, the direct co-culture of apoptotic cancer cells with macrophages induced significant changes in the phenotype, cytotoxicity, and cytokine secretion profile of macrophages (50). In another report, the direct co-culture of THP-1 cells with A431 human epidermoid cancer cells resulted in “educating” macrophages and altering their cytokines profile into a more pro-tumorigenic type (51). Experimental modalities for direct co-culture studies evolved in the last decade with a significant advance in the field of 3D engineered models, that have emerged as useful tools to fill the gap between conventional 2D systems and animal models (52). For example, microfluidic devices, that rely on the use of micron-sized channels to handle small fluid volumes have been used to recapitulate complex physiological microenvironments, thanks to their ability to control cellular, biochemical, and physical components. Microfluidic platforms have been successfully used to study cell migration, angiogenesis, and other cancer-associated phenomena, and are increasingly being leveraged to model cancer-immune cell interactions (53–55). Tumour spheroids are 3D cellular aggregates of uniform or heterogeneous cell types and they have emerged as promising *in vitro* platforms, useful not only for disease modelling but also for drug screening. Spheroids have been shown to mimic some histological features of human cancer: they have been used to recapitulate some key features of gliomas, such as their structural organization, hypoxic core and gradient distributions of oxygen (56, 57). Macrophages can be incorporated into tumour spheroids by directly forming spheroids from a mixed macrophage/cancer cell suspension. Alternately, macrophages have been reported to be able to infiltrate tumour spheroids, offering a more physiologically relevant method of macrophage incorporation (58, 59). Organoids are a new model system where complex multicellular structures of primary cells can be grown in a 3D matrix to recapitulate the biology of the parent tissue. This experimental model can be reliably generated by a wide variety of normal and cancerous tissues and offers several advantages including the ability to be genetically engineered and to be implanted *in vivo*. Furthermore, organoid cultures reproduce many features of their source tissue, including genetic and epigenetic alterations and drug sensitivity. In fact, organoids capture the cellular diversity, gene expression, and mutational profiles of their parental tumour of origins. Most notably, to obviate the limited expansion potential of tumor-immune infiltration, organoids can be co-cultured with autologous peripheral immune cells (60); macrophages can be directly incorporated into organoids during fabrication, allowing their infiltration into established organoids, or seeded into the surrounding matrix of embedded organoids (61). The generation of organoids from patient samples which can then be genetically engineered or co-cultured with autologous inflammatory cells ex vivo creates a uniquely powerful human system to study the role of inflammatory cells in driving cancer initiation and progression. Several aspects related to the role of TAMs in cancer growth, progression and therapy are still open issues:

- What is the potential of genetically engineered macrophages to displace the normal macrophages already present in hypoxic regions of tumors?
- What is the role of chemotaxis and chemokine production in the efficacy of TAMs when used as vehicles for drug delivery to hypoxic tumor sites?
- What are the underlying mechanisms in reeducating macrophages?
- What are the most important mechanisms of the interactions between TAMs and cancer cell plasticity?
- What are the primary factors of intracellular signalling between tumor cells and macrophages?
- How to define and understand the complex behavior of TAMs toward their interactions with other TME elements such as vasculature network, ECM, fibroblasts, and oxygen uptake?

In the next section, we will focus on the contribution of mathematical modelling in shedding light to these key questions.

## 3 Mathematical modelling of TAMS

Mathematical models have a long tradition in addressing oncology related questions and more recently focuses on immuno-oncology. In this section, we aim to review the mathematical models based on the key questions from the previous section. In particular here we review modelling studies that focus on: biological mechanisms of TAMs, macrophages as drug carriers, TAMs repolarization dynamics, tumor macrophage plasticity, and the role of TME elements (e.g. vasculature network, ECM, fibroblasts and oxygen uptake). In **Table S1**, we provide all the relevant mathematical models that incorporate the TAMs repolarization in models based on different aspects of TME and the major role of macrophages. In **Table S2**, we summarize the key elements and a brief overview of each article.

### 3.1 Macrophages as drug carriers

#### 3.1.1 Early studies of tumour-macrophage interactions

The earliest studies that considered macrophages in a mathematical model was referred in the series of works from Owen and Sherratt (62–64). They considered the killing ability of macrophages against the cancerous cells in a system of differential equation models to understand the tumour-macrophage mechanisms by assuming solely anti-tumoural macrophage effects. The main limitation of their models was the lack of accurate parameterization, due to the insufficient experimental data, and the absence of pro-tumoural functions by TAMs. Moreover, their models ignored the spatial structure induced by hypoxia and necrotic regions (62–64). Meanwhile, Griffith et al. (65) came up with the idea of arming macrophages with a therapeutic gene to affect gene-dependent enzyme prodrug therapy. They assumed that macrophages could infiltrate the tumors and can respond to the corresponding hypoxic microenvironment during the gene therapy (65). Therefore, mathematical models were developed to investigate the role of such infiltrating macrophages in tumors. Kelly et al. (66) proposed a model that described macrophage infiltration into small vascular tumors. They compared the simulated tumor infiltration of macrophages against their experimental data for chemoattractant-producing (HEPA-1) and chemoattractant-deficient (C4) spheroids. Spheroid’s size, spatial structure, and chemoattractant distribution were the main parameters to influence the rate of macrophages infiltration (66).

Owen et al. (67) developed a model of a growing avascular tumor spheroid where its volume is filled with tumor cells, macrophages, and ECM. They also considered the oxygen-dependent production of macrophage chemokine. They found out that chemotactic sensitivity is a key determinant of macrophage infiltration and tumor size (67).

Byrne et al. (68) used a model based on the growth of avascular tumor spheroid to evaluate the curing ability of engineered macrophages. In a simple, spatially uniform model, they assume genetically-engineered macrophages kill tumor cells wherein contact (68).

#### 3.1.2 Hypoxic regions as targets of macrophage-mediated drug delivery

Hypoxic areas are the hardest targeted regions for conventional drug therapy due to poor vascularization. Webb et al. (69) showed that effective targeting of hypoxic tumor cells by macrophages would be benefited by limiting-diffusivity or using non-cell-cycle dependent drugs. Their model was based on *in vitro* multi-cellular spheroid study (T47D tumor spheroids) for macrophage-based targeting that couples hypoxia-induced enzyme production with a pro-drug delivery at the tumor surface (69).

**Figure 2:**
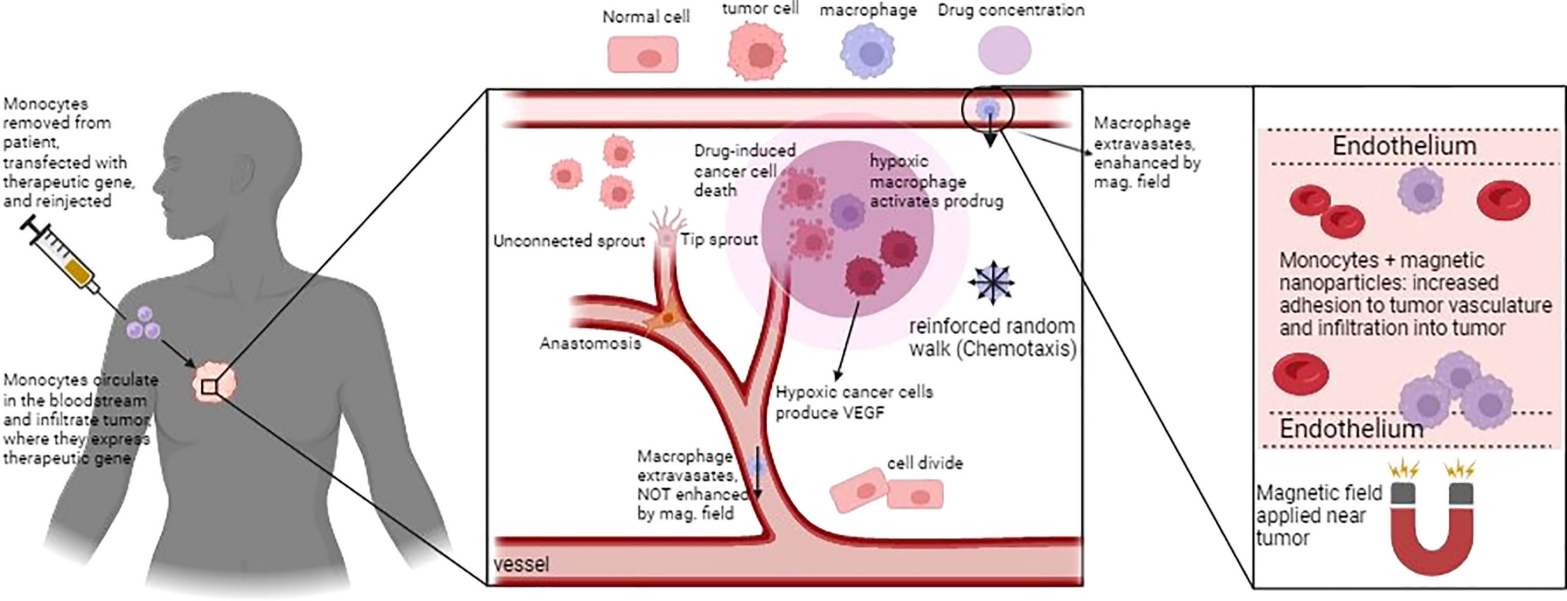
Schematic figure of macrophage-based cancer therapy and the corresponding mathematical model in Owen et al., (70). Macrophages can infiltrate and activate a pro-drug within the hypoxic region of tumor. The loaded macrophages, guided by the external magnetic field, extravasate in the tumour microenvironment to release their anti-tumoural action, Figure reproduced from reference (70).

Owen et al. (70) developed a spatio-temporal mathematical model of preloaded macrophages with nanomagnetic particles guided by an external magnetic force in a 2D vascularized tumor. They evaluated the ability of magnetically-guided macrophages in infiltrating the hypoxic region of the tumor to optimize treatment. Results predicted that the maximum anti-tumor effect occurred by the synergy of macrophage-based therapy and conventional chemotherapy. The timing was identified as a significant factor in this combined therapy since macrophage chemotherapy was administered shortly before the conventional chemotherapy. Projected results were drastically enhanced by using magnetic nanoparticles depending on the strong external magnetic field (70). **Figure 2** shows the whole idea of their works in designing a genetically engineered macrophage to eliminate the tumor cells at the different stages.

Leonard et al. (71) used the nanoparticle albumin-bound-paclitaxel (nab-PTX) encapsulated with the multistage vectors to target hypovascularized metastatic lesions. They employed macrophages to deliver this drug to the liver metastases derived from breast cancer. In their work, they combined experimental results with mathematical modelling. They evaluated their results by the *in vivo*, *in vitro*, and in silico results towards increasing the efficacy of nab-PTX therapy. This work could be also useful to assess how the mathematical models support results generated by the experimental models (71). Further, they investigated the therapeutic efficacy of nab-PTX as a function of macrophage phenotype and the ability of these nanoparticles to polarize TAMs towards M1 phenotype (72).

### 3.2 The effect of TAM repolarization

In this section, we reviewed models that investigate the macrophage phenotypic plasticity, as well as investigate the impact of TAM repolarization on tumor growth. These studies typically model the interactions between the macrophages and other microenvironmental elements such as cancer cells, stromal cells, and different immune cell types, to explore the role of macrophage repolarization on tumor growth. Macrophage repolarization has been recognized as a therapeutic strategy to switch protumor macrophages into anti-tumor macrophages with the ability to express tumoricidal activities.

#### 3.2.1 TAM repolarization with a linear transition rate

Louzoun et al. (73) investigated the interaction of macrophage-tumour cells in pancreatic cancer, which was able to induce polarization of pro-inflammatory M1 macrophages into protumoural M2 macrophages that undermine the immune response (73, 74). They considered both macrophage phenotypes in their model to explore how immunotherapy treatment could be optimized. The model described a temporal cancer-stromal-immune interaction of cells at different time scales, i.e., days for cell-cell interactions and minutes/hours for cytokine responses and considered a constant transition rate for macrophage polarization and re-polarization dependent on the rate of different cytokines, including TGF-*β*, IL6, MCSF, and GMCSF. Their model was validated on preexisting experimental data of Ellermeier et al. (75). The results showed that the feedback loop between the tumor cells, endothelial cells, and immune responses can significantly impact tumor growth dynamics. The model had been used to examine optimization strategies for pancreatic cancer treatment, particularly on the immune system’s role in chemotherapy. Their model assumed M2 and myeloid-derived suppressor cells (MDSC) reside in one compartment and did not consider the role of immune responses such as T regulatory cells and interactions between Th1, Th2, and Th17 (73). For better understanding the role and prognostic values of TAMs in pancreatic cancer, 76 provided a meta-analysis of 1699 patients in 13 studies (76).

Den Breems and Eftimie (77) also investigated the role of macrophage repolarization on tumor growth. This study developed a temporal mathematical model to encompass the interactions between immunogenic and non-immunogenic tumor cells, M1/M2 macrophages, and Th1/Th2 cells. They considered logistic growth for both tumor cells and macrophage proliferation. A linear constant rate was used for the switching between M1 and M2 phenotypes. The model was calibrated using the experimental data for mice melanoma (78). Stability analysis was performed to analyse the dynamic behavior of the system and to find out how model parameters affect it. This study was noteworthy for examining the interaction of macrophage phenotypes with other immune cell types within the TME, such as Th1 and Th2 cells. Finally, they estimated the effect of the M2/M1 ratio and the associated repolarization of macrophages on tumor growth and tumor decay dynamics (77).

#### 3.2.2 Macrophages as a continuous spectrum of phenotypes

**Figure 3:**
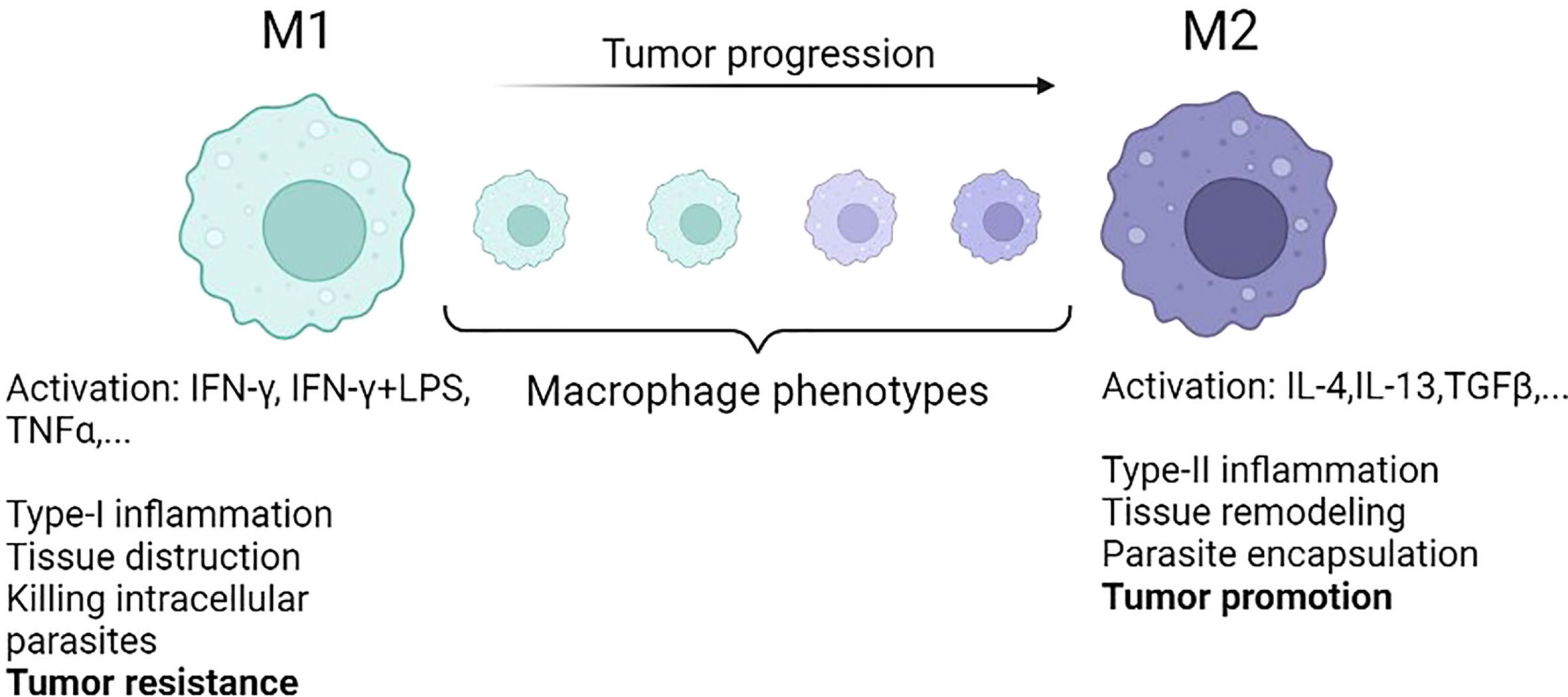
The conventional M1 and M2 macrophages represent the two extreme phenotypes represented in different models. In reality, during tumor progression, macrophages phenotypic transition is taking place from M1 to M2 in a continuous way [reproduced from reference (79)].

Departing from the M1/M2 paradigm, Eftimie (79) proposed a continuous phenotypic transition for the macrophage population in the context of tumor growth. In this study, the focus was on breast cancer (4T1 murine breast cancer cell line) and the goal was to compare the resulting tumour dynamics when including discrete M1-M2 or continuous TAMs phenotype assumption in the corresponding mathematical models. This study also considered the presence of TAMs during tumor dormancy. In terms of the steady-states, the switching rates of macrophages in the continuous model did not have any effects on the type and stability of model steady-states. The numerical simulations for both scenarios showed relatively similar dynamics. They also mentioned that the M2 to M1 repolarization could not solely lead to tumor elimination unless it was followed by an increased rate of phagocytosis of tumor cells induced by macrophages. Combined phagocytosis and direct repolarization of macrophages are more effective therapeutic approaches, resulting in a higher rate of phagocytosis (79). In a follow-up study, Eftimie and Barelle (80) extended this model to explore the tumor evolution in NSCLC involving three macrophage subpopulations, namely M1, M2, and mixed M1/M2 phenotypes. They assumed that macrophages can polarize and repolarize between the mixed M1/M2 macrophage phenotype. The model was parameterized according to experimental data derived from murine models. The results showed that the half-life of macrophages influenced the therapeutic outcomes and the macrophage repolarization treatment was influenced by the mixed phenotype of macrophages. Results emphasized the need for further research on macrophage experimental kinetics, specifically macrophages with mixed phenotypes (80). **Figure 3** shows the continuous and discrete forms of macrophage polarization in the TME based on their features.

#### 3.2.3 Tumor cell vs TAM plasticity

**Figure 4:**
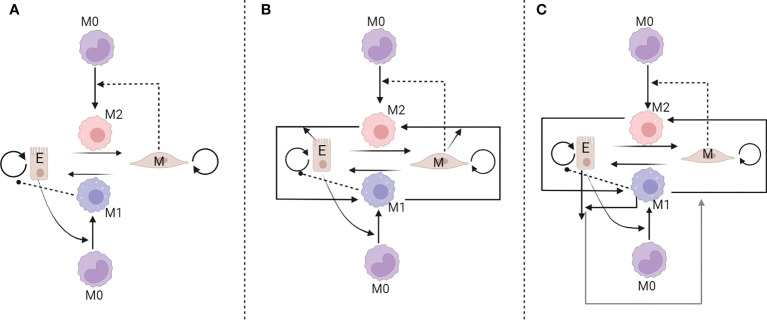
Three interaction networks developed to model the interplay between tumour and macrophages. In the model **(A)** epithelial cells polarize monocytes into M1-like macrophages, whereas mesenchymal cells polarize monocytes into M2-like macrophages. In models **(B, C)** additional feedbacks are assumed [reproduced from reference (83)].

Phenotypic plasticity has been considered as one of the new hallmarks of cancer in the latest version of the fundamental article of Hanahan (81). Despite being a therapeutic challenge, plasticity also reveals novel therapeutic targets that have, until recently, been overlooked (82). In this regard, Li et al. (83) developed a mathematical models (see **Figure 4**) to study the interaction between cancer cell phenotypes (epithelial and mesenchymal) and polarized macrophages (M1 and M2). Their study looks into the plasticity of both cancer cells and macrophages. They use a sample of the four TCGA datasets to calculate the correlation coefficients for epithelial to mesenchymal transition (EMT) scores to validate their model predictions. A three sets of ordinary differential equation system was developed and their analysis showed that the existence of multi-stability is due to the interactions between tumor cells and macrophages. In particular, two distinct cancer phenotypes assumed to play a crucial role in interconversion between M1 and M2 macrophages; mesenchymal cells enhance the M1 to M2 transition, while epithelial cells support M2 to M1 transition. Moreover, their model suggested a therapeutic strategy for maintaining the system in a M1-dominated and cancer-free steady-state by considering the changes in switching rate from mesenchymal to epithelial cells and lowering the growth rate of mesenchymal cells. However, their model lacks the spatial features of the aforementioned interactions and no migration factors induced by epithelial cells to EMT. It should be noted that EMT and macrophage polarization were considered binary mechanisms and not a spectrum of states (83).

### 3.3 Multi-scale approaches towards the macrophage-based treatments

Mahlbacher et al. (84) developed a multi-scale model based on Macklin et al. (85) and Wu et al. (86), where the tumor growth, vascular network, intravascular blood flow, and tumor progression were taken into account (85, 86). In their model, they considered three tumor-associated macrophages subtypes, namely, M1, M2, and Tie-2 expressing macrophages (TEM) variants. A 2D grid of vascularized network model was implemented to investigate the recruitment of macrophages as well as the spatial dynamics of other TME components, such as cytokine transport. The mechanisms involved in their model consist of M1 releasing nitric oxide (NO), M2 releasing growth-promoting factors, and TEM promoting M2 production and facilitating tumor-induced angiogenesis, *via* secretion of Angiopoietin-2. The simulation results show that M2 is enough to promote tumor growth without the assistance of the TEM population. Therefore, TEM ablation in immunotherapy does not help to reduce tumor growth in the presence of a significant amount of M2 in TME. The overall outcome of their model could be potentially useful in cancer immunotherapy optimization (84).

Leonard et al. (87) explored the impact of macrophage phenotypes ratio and the associated macrophage polarization by employing the CRISPR system in a controlled *in vitro* setting applied to breast cancer liver metastasis. Their model was based on the assumption of “agent affecting macrophage polarization” and focused on the macrophage polarization in the vicinity of TME. A corresponding nano-therapy was explored against the *in vitro* breast tumor growth. The results showed that the M2-tumor interaction might have a dual effect in suppressing and promoting tumor growth. Moreover, their study showed that the polarization of macrophages could be a significant parameter for establishing combined therapeutic regimens (87). It is worth mentioning that their study is solely focused on metastatic TME while other aspects of TME such as T-cell exhaustion and enrichment of MDSC were not considered (87).

Suveges et al. (88) investigated the macrophage re-polarization (M2 to M1) with a multiscale moving boundary framework to explore its dependency on the tumour leading edge and within a determined time-scale relevant to cancer immunotherapy. For this reason, they developed a model formerly implemented in Trucu et al. (89) and Shuttleworth and Trucu (90). The tumor interface was the main focus of the region to show the dynamics of M1 and M2 cells. The dependency of re-polarization to both spatial and temporal was investigated. They concluded that additional strategies to target the tumor spread or tumor stroma were needed to fully stop the cancer progression.

#### 3.3.1 Modelling tumor macrophage intercellular communication

Tumor cells and macrophages can communicate with each other with a set of chemical signalling pathways, mainly EGF and CSF-1. Tumors cells secrete CSF-1 to activate macrophages in secreting EGF and subsequently promote tumor growth (91, 92). Knútsdóttir et al. (2014) investigated paracrine and autocrine signalling loops associated with the role of macrophages in facilitating the metastatic process in breast cancer. They addressed questions regarding the conditions of sufficient paracrine loop to generate aggregation of tumor cells and macrophages, the effect of drugs on treatment, signalling dynamics, and the size dependency of an aggregate on signalling parameters. First, they developed a partial differential equation (PDE) system to explore the impact of the paracrine signalling loop between TAMs and tumor cells, with respect to the tumor cell aggregation. They considered the density of macrophages, tumor cells, CSF-1, and (EGF) as the main four variables. Then, a cell-based discrete 3D simulation was developed to take into account the single-cell migration dynamics. They revealed that the signalling parameters impact the cellular collective behavior in terms of aggregation. Moreover, they compared the mean-field PDE model and the detailed individual-cell behaviors. Their model was mainly used to understand how autocrine signalling could impact cell proliferation and accordingly design new drugs. They provided scenarios of tumor reduction or elimination by decreasing the secretion of CSF-1, EGF, the density of macrophages, chemotaxis sensitivity, and increasing the degradation of CSF-1 or EGF. Interestingly, macrophages were not considered the main target cells in their model and there was no macrophage polarization in their model (92). In a further study, they analyzed the aggregation of cells in 2-D to predict the migration patterns and provided qualitative agreement with the *in vitro* and *in vivo* experimental studies, especially regarding the role of the paracrine signalling loop in cancer cell invasion (93).

#### 3.3.2 Interactions of TAMs and TME elements

To better understand the complex behavior of TAMs and tumor-macrophage interactions, various studies considered different aspects of TME such as microvessels, nutrients, fibroblasts, and the ECM. In this regard, Wells et al. (94) developed a hybrid discrete-continuous agent-based model for a nascent metastatic TME. Their tumor model involved the key processes of vascularization, oxygen uptake, angiogenesis, and macrophage infiltration. Macrophages were assumed to be equipped with chemotaxis, polarization and be involved in tumor killing. TME was characterized by the early days of tumor development. At each lattice site, naïve macrophages were recruited in a probabilistic manner. A central feature of this study was the implementation of a system-wide multi-parametric sensitivity analysis to identify useful metrics for the analysis of such tumor-immune interaction models. This model was intended to improve immunological control of early tumors and facilitate the engineering of cell-based therapies to tackle immune dysfunction. Biological mechanisms such as tumor mutation, phenotypic evolution, tumor chemotaxis, and escalating invasion, were neglected due to their relevance at the longer time scales (94).

Norton et al. (95) used an agent-based model to investigate the interactions of triple-negative breast vascularized tumor with its microenvironment. Their model included breast cancer stem cells, cancer progenitor cells, endothelial cells, macrophages, and fibroblasts. Breast cancer cells form “invasive fingers” which were accompanied by the presence of macrophages. They showed that macrophages support tumor cell migration/metastasis and the presence of macrophages in the invasive zone is associated with tumor migration dynamics. They showed that such invasive tumor cells cannot last long without the recruitment of macrophages, which increased in tumor growth rate. However, the increase in macrophage infiltration did not change the overall tumor growth. The limitation of their work was that they consider macrophages as a single population of TAMs in their models and did not consider the effects of symmetric stem cell division and immune response to the tumor (95).

#### 3.3.3 The interaction between TAMs and T-cells

One of the earliest works regarding the role of macrophage and T lymphocyte interactions is the article of 96. They developed a model based on cytotoxic T lymphocytes, macrophage antigen presentation, activation of T-helper cells, and production of lymphoid factors. In this article, three different macrophage functions were considered, namely macrophages without antigens and activating factors, macrophages loaded with antigens, and cytotoxic macrophages. However, it was assumed that cytotoxic macrophages are activators of T-helper cells and producers of IL-1. Their result showed that the amounts of tumor antigenicity could impact the eradication induced by the corresponding effector cells. However, this study could just be considered as a theoretical approach due to the lack of experimental data and biologically relevant parameters (96). Curtis et al., 2020 considered the macrophage and T-cell interactions in the context of metastatic TME. They used a modified mathematical model, coupled with the macrophage model, which first appeared in 85 and 86 (85, 86). They considered a small metastatic tumour able to produce angiogenic factors and immune chemo-attractants, where the latter enhance immune cell migration towards the TME. The model included an adaptive immune response, namely *CD*8^+^ cytotoxic T cells, *CD*4^+^ Th1 cells, and *CD*4^+^ Th2 cells in a 2D vascularized network grid with hypoxic and proliferating tumor regions. They assumed different M1:M2 ratios, based on various stages of the cancer, and analyzed the resulting behavior of the system. The result showed that a higher value of M1:M2 decreased the tumor ratio and increased the number of immune cells in TME. However, this decrease cannot induce tumor regression due to the saturated number of Th1 cells at higher ratios of M1:M2. This model could include further TME parameters such as cancer-associated fibroblasts (CAFs) and ECM as well as other myeloid immune cells to provide a customized immunotherapeutic regimen to different patient-specific conditions (97). Cess and Finley (98) investigated the interactions between T-cells, macrophages, and tumor cells on how the immune response changes along with three macrophage-based immunotherapy schemes, namely macrophage depletion, recruitment inhibition, and macrophage reeducation. They developed a hybrid multi-scale agent-based model to compute the intracellular signalling mechanisms in their model. A neural network model was employed to reduce the computational costs and complexity of the model. Using the parameter values that were found in previous modelling and experimental studies, it was found that macrophage reeducation is the best immunotherapy strategy due to its strong role in T-cell induction. Moreover, they showed that the tumor proliferation and macrophage recruitment rate could be two major parameters in immunotherapy efficacy. Their model depicted only a general form of tumor behavior and neglected nutrient uptake or hypoxia as an effective parameter in promoting M2 differentiation or T-cell suppression (98).

#### 3.3.4 Interactions between ECM and TAMs

In recent years the ECM has received considerable attention due to its role in tumor expansion dynamics and impact on therapies. During cancer progression, the ECM undergoes remodeling *via* synthesis and degradation. ECM plays a crucial role in the evolution of cancer and response to the therapies. Apart from the cancerous cells, macrophages can also degrade the ECM by expressing matrix metalloproteases (MMPs). Hudson et al. (99) investigated the interactions between macrophages, ECM, and tumor cells to understand the behavior of tumor growth in the context of metastatic liver cancer (post alcoholic liver disease). They considered primary and metastatic tumor types along with two distinct ECM, namely normal and hepatic ECM (transitional). Their simulations involved naïve and polarized macrophages for both tumor types and ECM types. Naïve macrophages are typically extravasated from the vasculature network. TNF-α and TGF*β* were considered cytokines that influence the polarization of M1 and M2, respectively. The results showed that the primary tumor had more distributed M1 and M2 subtypes. On the other hand, metastatic tumors had a higher M1 concentration. The macrophage ratio was also relevant to the final tumor size. Metastatic tumor radius was smaller than the primary tumor in both normal and hepatic ECM (99).

Moreover, Suveges et al. (100) added the effect of ECM for both fibre and non-fibre components accompanied by the presence of M2 macrophages to investigate the movement of M2-like macrophages on the ECM remodeling and the corresponding collective behavior of cancer cells (100). The tumor interface dynamics was the main focus of this study. Macrophage polarization mechanisms were investigated along the presence/absence of nutrients. The nutrients influenced both proliferation and death of the macrophages and cancer cells. The results showed that macrophage re-polarization approaches might be accustomed to the TME and the ECM degradation level. This study focused only on the qualitative dynamics of the system through its non-dimensionalized model analysis. Further biological relevance could be introduced, for instance by modelling the effect of the vascular network in their models (88).

## 4 Discussion

Herein we review models addressing questions related to the role of TAMs in tumour growth dynamics. In particular, researchers have investigated therapeutic strategies using different TAM properties such as migration towards hypoxic regions, switching between anti- and pro-inflammatory phenotypes and interaction with various immune cell types or other entities in the TME. Although the reviewed literature addresses a large number of questions and related issues, some challenges are still open.

One of the important challenges regarding mathematical modelling is related to the complexity of the developed models. Typically, modelers either design simple and low-dimensional models focusing on selected biological mechanisms or attempt to exploit all available biological knowledge and build complex models. The former models involved low number of parameters, which could be typically quantified. However, these parameters might suffer identifiability and represented more underlying processes rather than the intended ones, e.g. an *in vivo* proliferation rate did not represent only the cell’s intrinsic division rate but also other microenvironmental processes such as nutrient availability, mechanical stresses related to cell division etc. On the other hand, in complex models the parametrization problem was more intense since the large number of parameters makes their calibration difficult. The right balance of modelled biological mechanisms and the design of specialised experimental assays may allow for improved parameter calibration and identification.

In addition to the parametrization challenge was the lack of precise knowledge of the involved biological mechanisms. In particular, cancer biology and immunology are research intensive fields, where a plethora of biological mechanisms remained to be discovered. As a result, developing a model at the best case involves assumptions of phenomenological terms that account for this lack of knowledge. Therefore, quantitative and personalised predictions are a daunting task. Meanwhile, in order to overcome these problems, data-driven techniques combined with mechanistic modelling can improve the prediction accuracy of the models (6, 101). Such methods have the potential to bring mathematical closer to the clinical practice.

The exact details of macrophage phenotypic plasticity dynamics are partially known. Biologists know how to polarize macrophages in their extreme phenotypes using pro- and anti-inflammatory cues. However, it remains elusive what is the time that an M1 needs to become M2 under anti-inflammatory conditions and vice versa. Also what is the role of other microenvironmental entities, such as other immune cells, ECM, fibroblasts (102, 103). In mathematical terms, what are the type of bifurcations that macrophage phenotype undergoes when varying different combination of microenvironmental elements. Accordingly, what is the feedback of macrophage plasticity to the surrounding cells. Designing such experiments is cardinal in shedding light to the complexity of the underlying biology. The latter will facilitate the development of more biologically and clinically relevant models.

Finally, a few words regarding the future of mathematical modelling of TAMs and tumour growth dynamics. As stated above, the system consists of two interacting plastic populations, TAMs and cancer cells. For clinically relevant tumour growth and progression predictions involving the impact of TAMs, it is pivotal to develop ways that circumvent our biological knowledge limitations (104). Apart from the afore-mentioned integration of machine learning and mechanistic modelling, an interesting perspective is the identification of cell-decision making principles, e.g., based on the Least Environmental Uncertainty Principle (LEUP) (105, 106). Since phenotypic plasticity is a type of cell-decision making, such principles can provide a unifying framework for understanding the underlying biology, modelling phenotypic changes of heterogeneous cell populations and for developing novel prediction algorithms. Because identifying such organization principles is extremely intriguing, further research is required to be useful in a clinical setting.

## Author contributions

PS and FM are both co-first authors and contributed equally. PS and HH conceived the idea to review all the relevant mathematical models in the context of tumor-macrophage interactions and wrote and revised the manuscript. FM and ML conceived and wrote the biological sections and revised the manuscript. AD reviewed the manuscript and provided useful comments. HH and ML supervised the work. All authors provided critical feedback and helped shape the review, analysis, and manuscript. All authors contributed to the article and approved the submitted version.

## Funding

PS and HH have received funding from the Bundesministerium für Bildung, und Forschung (BMBF) under grant agreement No. 031L0237C (MiEDGE project/ERACOSYSMED). Moreover, HH and ML would like to acknowledge the support of the Volkswagenstiftung for “Life?” initiative (96732). HH acknowledges the support of the FSU grant 2021-2023 grant from Khalifa University. FM and ML have received funding from Ministero della Salute (MiEDGE project/ERACOSYSMED; Bando Ricerca Finalizzata RF-2019-12371549).

## Acknowledgments

Finally, we thank the Centre for Information Services and High Performance Computing at Technische Universität Dresden for providing high-performance computing infrastructure.

## Conflict of interest

The authors declare that the research was conducted in the absence of any commercial or financial relationships that could be construed as a potential conflict of interest.

## Publisher’s note

All claims expressed in this article are solely those of the authors and do not necessarily represent those of their affiliated organizations, or those of the publisher, the editors and the reviewers. Any product that may be evaluated in this article, or claim that may be made by its manufacturer, is not guaranteed or endorsed by the publisher.
