# Supplementary material for: The impact of tumor associated macrophages on tumor biology under the lens of mathematical modelling: A review
Source: Front Immunol. 2022 Nov 10;13:1050067. doi: 10.3389/fimmu.2022.1050067 (PMC9685623; doi:10.3389/fimmu.2022.1050067)
Supplement: Supplementary file 1 [file DataSheet_1.pdf]

# Supplementary Material

## 1 SUPPLEMENTARY DATA

Table S1 summarises all of the literature discussed in this review. We present key features of each model based on the kind of cancer, macrophage phenotypes, hypoxia involvement, tumour vasculature, other types of immune cells, and the role of macrophages in model. Table S1 is organised according to the role of macrophages in respective models.
Table S2 was designed to provide a quick overview of each piece of literature included in the main review paper. Here you may discover the important subject of each study, which includes a summary of the main points, the model type, scale of the model, clinical usage, data type.

**Table S1** Shows different applications of mathematical models in the context of Tumor-immune interactions. There are three main categories as macrophages works as drug carriers, the biological mechanism of macrophages in tumor, and macrophages as targeted cells. The studies are sorted based on the role of macrophages in the model.

| Study | Type of cancer | Macrophage phenotypes | Model associated with hypoxia | Angiogenesis | Other immune cell types interaction | Role of macrophages in the model |
|---|---|---|---|---|---|---|
| De Boer et al. (1985) | Non-specific | - | - | - | T lymphocyte and T-helper cells | macrophages as activators of T-helper cells and producers of IL-1 |
| Owen and Sherrat (1997, 1998, 1999) | Non-specific | - | - | - | - | Macrophage infiltration in tumor |
| C.E.Kelly et al. (2002) | hepa-1 and C4 spheroids | - | ✓ | - | - | Macrophage infiltration in tumor |
| Owen et al. (2004) | T47D tumor spheroids | - | ✓ | - | - | Macrophages as drug carriers |
| Byrne et al. (2004) | Non-specific | - | - | - | - | Macrophages as drug carriers |
| Webb et al. (2007) | T47D tumor spheroids | - | ✓ | - | - | Macrophages as drug carriers |
| Owen et al. (2011) | Non-specific | - | ✓ | ✓ | - | Macrophages as drug carriers |
| Leonard et al. (2016) | Metastatic TME in the liver | - | ✓ | ✓ | - | Macrophages as drug carriers |
| Louzoun et al. (2014) | Pancreatic cancer | ✓ | - | - | CTL cells | Macrophage re-polarization |
| Den Breems and Eftimie (2016) | Melanoma (Mice) | ✓ | - | - | Th1/Th2 cells | Macrophages re-polarization |
| Curtis et al. 2020 | Liver metastatic cancer | ✓ | ✓ | ✓ | T-cells | macrophage repolarization ratios |
| Leonard et al. (2020) | Breast cancer Liver metastasis | ✓ | ✓ | ✓ | - | Macrophage re-polarization |
| Cess and Finley (2020) | Non-specific | ✓ | - | - | T-cells | macrophage re-polarization and other immuno therapeutic strategies |
| Eftimie (2020) | Murine breast cancer | ✓ | - | - | - | Macrophage re-polarization |
| Eftimie and Barelle (2021) | NSCLC (Murine data) | ✓ | - | - | Mixed M1/M2 phenotype | Macrophage re-polarization |

| | | | | | | |
|---|---|---|---|---|---|---|
| Suveges et al. (2022) | Non-specific | ✓ | ✓ | - | - | Macrophage re-polarization |
| Knútsdóttir H, et al. (2014, 2016) | Breast cancer | - | - | - | - | Indirect effect of macrophages in the system |
| Wells et al. (2015) | Nascent metastatic TME | ✓ | ✓ | ✓ | - | Indirect effect of macrophages in system |
| Mahlbacher et al. (2018) | Metastatic TME in the liver | ✓ | ✓ | ✓ | Tie2-expressing macrophages TEM | Indirect effect of macrophages in system |
| Li et al, (2018) | Non-specific | ✓ | - | - | - | Indirect effect of macrophages in system |
| Norton et al. (2018) | Triple negative breast cancer | - | ✓ | ✓ | - | Indirect effect of macrophages in system |
| Hudson et al. (2019) | liver metastasis post hepatic injury | ✓ | ✓ | ✓ | - | indirect effect of macrophages in the system |
| Suveges et al. (2020) | non-specific | ✓ | - | - | - | macrophage infiltration in tumor |

**Table S2** shows all the provided studies by considering the key subject, model type, scale, clinical usage, and type of data used for the study.

| study | Key subject | Model type | Scale | Clinical usage | Type of data |
|---|---|---|---|---|---|
| De Boer et al. (1985) | presenting a model of macropages and T lymphocyte to generate an anti-tumour response | ODE | Tissue-scale | The authors did not mention any clinical benefits from their model | Data estimation and using the previous literature |
| Owen and Sherrat (1997) | Existence of travelling wave solutions in the macrophage-tumor interactions | PDE | Tissue-scale | The authors did not mention any clinical benefits from their model. | Theoretical studies on avascular tumor |
| Owen and Sherratt (1998) | developing a model for the role of macrophages in avascular tumor growth | ODE | Tissue-scale | The authors did not mention any clinical benefits from their model. | Theoretical studies on avascular tumor |
| Owen and Sherratt (1999) | They demonstrated spatio-temporal irregularities in the model solution | PDE | Tissue-scale | The authors did not mention any clinical benefits from their model. | Qualitative but validated with observations of Breast cancer cell-line |
| Kelly et al. (2002) | macrophage infiltration into avascular tumors or spheroids | PDE | Tissue-scale | it is a starting point in helping for finding the effective treatment to control tumor growth | using experimental data from breast cancer cell-line |
| Byrne et al. (2004) | Predict how the macrophages should be engineered and in what quantities to minimise the tumour burden. | ODE | Tissue-scale | early study for the use of genetically engineered macrophages to treat cancer | Qualitative information |
| Owen et al. (2004) | Study on the abilities of engineered macrophages to displace the normal macrophages already present in hypoxic regions of tumours | PDE | Tissue-scale | Engineered macrophages could be delivered | HEPA-1 spheroids in-vitro data |

| | | | | | |
|---|---|---|---|---|---|
| Webb et al. (2007) | To find out the comparison of the responses of avascular tumor spheroids in two modes of action. | PDE (free boundary problem) | Tissue-scale | They concluded that effective targeting of hypoxic tumour cells may require the use of drugs with limited mobility or whose action does not depend on cell proliferation | using multi-cell spheroids data |
| Owen et al. (2011) | Effects of macrophage-based gene therapy targeting on hypoxic tumor regions | PDE (2D) | Multi-scale | design and maximize the efficacy of combined therapeutic approaches in cancer. | in-vitro and in-vivo experimental data |
| Louzoun et al. (2014) | Roles of the state of the immune system in drug treatment. | ODE | Multi-scale | Immuno-modulatory drugs are effective in a narrow window of immune responses. | Data estimation and using the previous literature |
| Knútsdóttir H, et al. (2014, 2016) | Study the motility of breast cancer cells in an environment with the presence of macrophages by considering the autocrine and pracrine signalling loops | Sets of PDEs and discrete cell based model | Multi-scale | some of their results could be helpful in drug design (i.e anti CSF-1) for cancer immunotherapy | Experimental literature to quantify their parameters |
| Wells et al. (2015) | Studying the early stage of TME and its interaction with macrophages | hybrid discrete-continuous (HDC) agent based model | Multi-scale | Evaluation of potential engineered cell-based therapy strategies and using sensitivity analysis and establishing useful metrics | Data estimation and using the previous literature |
| Leonard et al. (2016) | Roles of the state of the immune system in drug treatment. | ODE | Multi-scale | Immuno-modulatory drugs are effective in a narrow window of immune responses. | Data estimation and using the previous literature |
| Den Breems and Eftimie (2016) | Interaction of Th1/Th2 and M1/M2 polarisation to control tumor growth | ODE | Tissue-scale | The ratio of M2/M1 could be used as a biomarker for decisions regarding various long-term patient treatments | Using Xenograft data |

| | | | | | |
|---|---|---|---|---|---|
| Mahlbacher et al. (2018) | How macrophages and fibroblasts interactions with the tumor microenvironment may affect tumor progression | hybrid model | Multi-scale | Establish the initial feasibility of a mathematical framework that could help to optimise cancer (macrophage) immunotherapy. | Breast cancer cell lines based on their previous studies |
| Norton et al. (2018) | using the model to investigate the effect of macrophage infiltration to the tumor growth | hybrid model | Multi-scale | Establish the initial feasibility of a mathematical framework that could help to optimise cancer immunotherapy. | Breast cancer cell lines (MB-231) |
| Li et al, (2018) | How macrophages of different polarisation (M1 vs. M2) can interact with Epithelial-Mesenchymal plasticity of cancer cells. How cancer cells exhibiting different phenotypes (Epithelial vs. Mesenchymal) can influence the polarization of macrophages. | ODE | Tissue-scale | Their results may help inform efficient therapeutic strategies. | Breast cancer cell line in liver (Metastatic microenvironment) and validated with gene-expression levels |
| Hudson et al. (2019) | Investigate the role of ECM (normal and injured) interacted with Naive and polarised macrophages | Hybrid model | Multi-scale | Bring therapeutic strategies to minimise liver tumor growth | Experiments with mouse model |
| Curtis et al. 2020 | M1:M2 ratio have an important role in tumor regression | Hybrid model | Multi-scale | Establish a modelling framework to analyse macrophage and T lymphocyte interactions in the TME | Parameters were fitted with their previous studies |

| | | | | | |
|---|---|---|---|---|---|
| Leonard et al. (2020) | The interaction between the nanoparticle-mediated chemotherapy in conjunction with macrophage polarisation | Hybrid model | Multi-scale | The results here show that the polarisation of macrophages may play an important role in the planning of combinatorial therapeutic regimens | Experimental results |
| Eftimie (2020) | The impact of changes in macrophages polarisation/re-polarisation rates, either induced by normal tumor progression or by external treatment. | coupled ODE-PDE | Tissue-scale | A combined therapeutic approach involving both a M2→M1 re-polarisation as well as an increase in phagocytosis might improve the therapeutic outcomes. | 4T1 murine breast cancer cell line. |
| Suveges et al. (2020) | Investigating of the directional movements of the TAMs on the tumour progression and interaction with ECM | Hybrid model | Multi-scale moving boundary | Macrophage re-polarisation in cancer immunotherapy | based on the literature data |
| Cess and Finley (2020) | How the immune response changes due to three macrophage-based immunotherapy strategies | Agent-based model and neural network | Multi-scale | Effects of continuous and cycling treatment on immune response and tumor growth. | based on the literature data |
| Eftimie and Barelle (2021) | How macrophages with a mixed phenotype impact on the lung tumors | ODE | Tissue-scale | The model could be beneficial in understanding the directionality of M2 dynamics in tumour boundary | based on the literature data |
| Suveges et al. (2022) | How macrophage re-polarisation depends on the time and space and how it affects the cancer development | Hybrid model | Multi-scale | Macrophage re-polarisation in cancer immunotherapy | based on the literature data |
